# Biological constraints limit the use of rapamycin-inducible FKBP12-Inp54p for depleting PIP_2_ in dorsal root ganglia neurons

**DOI:** 10.1186/1477-5751-12-13

**Published:** 2013-09-08

**Authors:** Jaeda C Coutinho-Budd, Samuel B Snider, Brendan J Fitzpatrick, Joseph E Rittiner, Mark J Zylka

**Affiliations:** 1 Curriculum in Neurobiology, University of North Carolina at Chapel Hill, Chapel Hill, NC 27599, USA; 2 Department of Cell Biology & Physiology, UNC Neuroscience Center, University of North Carolina at Chapel Hill, NRB 5109D, CB #7545, 115 Mason Farm Road, Chapel Hill, NC 27599, USA

**Keywords:** Phosphatidylinositol 4,5-bisphosphate, PIP2, Rapamycin, Inp54p, FKBP12, Dorsal root ganglia

## Abstract

**Background:**

Rapamycin-induced translocation systems can be used to manipulate biological processes with precise temporal control. These systems are based on rapamycin-induced dimerization of FK506 Binding Protein 12 (FKBP12) with the FKBP Rapamycin Binding (FRB) domain of mammalian target of rapamycin (mTOR). Here, we sought to adapt a rapamycin-inducible phosphatidylinositol 4,5-bisphosphate (PIP_2_)-specific phosphatase (Inp54p) system to deplete PIP_2_ in nociceptive dorsal root ganglia (DRG) neurons.

**Results:**

We genetically targeted membrane-tethered CFP-FRB^PLF^ (a destabilized FRB mutant) to the ubiquitously expressed Rosa26 locus, generating a Rosa26-FRB^PLF^ knockin mouse. In a second knockin mouse line, we targeted Venus-FKBP12-Inp54p to the Calcitonin gene-related peptide-alpha (CGRPα) locus. We hypothesized that after intercrossing these mice, rapamycin treatment would induce translocation of Venus-FKBP12-Inp54p to the plasma membrane in CGRP^+^ DRG neurons. In control experiments with cell lines, rapamycin induced translocation of Venus-FKBP12-Inp54p to the plasma membrane, and subsequent depletion of PIP_2_, as measured with a PIP_2_ biosensor. However, rapamycin did not induce translocation of Venus-FKBP12-Inp54p to the plasma membrane in FRB^PLF^-expressing DRG neurons (*in vitro* or *in vivo*). Moreover, rapamycin treatment did not alter PIP_2_-dependent thermosensation *in vivo*. Instead, rapamycin treatment stabilized FRB^PLF^ in cultured DRG neurons, suggesting that rapamycin promoted dimerization of FRB^PLF^ with endogenous FKBP12.

**Conclusions:**

Taken together, our data indicate that these knockin mice cannot be used to inducibly deplete PIP_2_ in DRG neurons. Moreover, our data suggest that high levels of endogenous FKBP12 could compete for binding to FRB^PLF^, hence limiting the use of rapamycin-inducible systems to cells with low levels of endogenous FKBP12.

## Background

The immunosuppressant macrolide, rapamycin, induces the dimerization of two naturally occurring protein domains: FK506 Binding Protein 12 (FKBP12) with the FKBP Rapamycin Binding (FRB) domain of mTOR [1]. These domains can be attached to other proteins to temporally and spatially control cell signaling with rapamycin or rapamycin analogs. For example, these domains were used to control cell growth and cell death [2], to translocate proteins to the plasma membrane or nucleus [3-5], and induce G protein-coupled receptor (GPCR) signaling [6].

Additionally, two groups used these domains to directly and selectively deplete the lipid PIP_2_ in cultured cells [3,4] and show that PIP_2_ was important for GPCR signaling and ion channel function [7-9]. Both groups used 1) a plasma membrane-anchored FRB domain and 2) a cytosolic PIP_2_-specific phosphatase (yeast Inositol polyphosphate 5-phophatase (Inp54p) or mammalian type IV 5-phosphatase) fused to FKBP12. In cell lines transfected with both of these components, rapamycin promoted dimerization of the FRB domain with FKBP12, and induced rapid translocation of the phosphatase to the plasma membrane where it hydrolyzed PIP_2_. PIP_2_ hydrolysis was visualized with a biosensor containing the pleckstrin homology (PH) domain of PLC∂1 (PLC∂1-PH) fused to a fluorescent protein [10-12]. This biosensor dissociates from the plasma membrane and enters the cytosol when PIP_2_ is hydrolyzed to phosphatidylinositol 4-phosphate (PI(4)P) and inorganic phosphate.

To date, this rapamycin-inducible system has been used in cell lines. Given the widespread importance of PIP_2_ in signaling and ion channel function [8,13,14], we hypothesized that this system, if adapted for use in animals, could also shed light on how alterations in PIP_2_ affect animal physiology and behavior. For example, PIP_2_ modulates Transient Receptor Potential (TRP) ion channels involved in heat and cold sensation, including TRPV1 and TRPM8 [15-21]. Moreover, we recently found that thermosensation and nociceptive sensitization could be reduced by indirectly decreasing PIP_2_ concentration in DRG [22].

Here, we sought to directly and selectively reduce PIP_2_ concentration in the plasma membrane of nociceptive DRG neurons to study the *in vivo* importance of PIP_2_ in regulating thermal sensitivity and nociceptive sensitization. To accomplish this goal, we knocked FKBP12-Inp54p fused to a variant of yellow fluorescent protein (Venus) into the CGRPα locus. CGRPα is a marker of peptidergic sensory neurons, a subset of which expresses the thermosensor TRPV1 [23,24]. We generated a second mouse containing a CFP-tagged, membrane-tethered FRB domain knocked into the ubiquitously expressed Rosa26 locus. By crossing both of these mice together, we were able to express both components of the PIP_2_ phosphatase system in peptidergic, small diameter DRG neurons and evaluate the performance of this system *in vitro* and *in vivo*. Unfortunately, we found that Venus-FKBP12-Inp54p did not translocate to the plasma membrane in DRG neurons following rapamycin treatment. Furthermore, our data suggests that a biological constraint—namely high levels of endogenous FKBP12—limits translocation in murine DRG neurons.

## Results

### Rapamycin induces translocation of Venus-FKBP12-Inp54p from the cytoplasm to plasma membrane in cell lines

Before generating knockin mice, we set out to verify that the rapamycin-inducible phosphatase components functioned in our hands as described [3,4]. For these experiments, we modified the FRB-CFP construct described in Varnai et al. (2006) by replacing the native FRB domain with the destabilized FRB^PLF^ mutant [25,26] to generate FRB^PLF^-CFP. This mutation confers greater sensitivity to rapamycin analogs, like C20-Marap, that can be used *in vivo*[25,26]. This construct also contains the palmitoylation sequence of human growth associated protein 43 (GAP43), a sequence that promotes plasma membrane localization in cell lines and DRG neurons [4,27]. Additionally, we replaced CFP in the yeast Inp54p construct described in Suh et al. (2006) with a yellow fluorescent protein (Venus) to permit simultaneous visualization of Venus-FKBP12-Inp54p and FRB^PLF^-CFP in live or fixed cells. The yeast phosphatase was chosen so that it could be immunologically distinguished from endogenous mouse 5-phosphatases.

**Figure 1:**
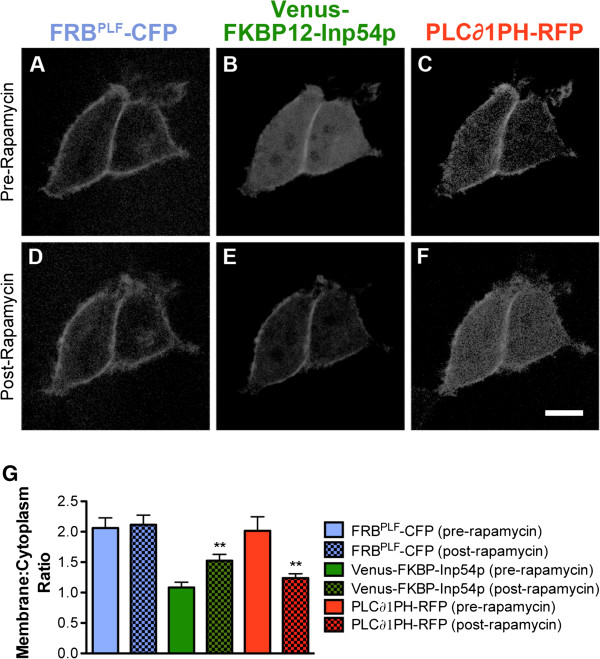
**Inp54p translocates to plasma membrane in HEK293 cells, reducing PIP**_**2 **_**biosensor levels following rapamycin treatment. A-F)** HEK293 cells were transfected with plasmids encoding FRB^PLF^-CFP, Venus-FKBP12-Inp54p and PLC∂1PH-RFP, a PIP_2_ biosensor. One day later, cells were imaged by live-cell confocal microscopy. Before rapamycin treatment, **(A)** FRB^PLF^-CFP, **(B)** Venus-FKBP12-Inp54p, and **(C)** PLC∂1PH-RFP were properly localized to the **(A,C)** membrane or **(B)** cytoplasm. While localization of FRB^PLF^-CFP remains constant, rapamycin (1 μM) induced rapid (<3 s) translocation of **(E)** Venus-FKBP12-Inp54p to the plasma membrane and **(F)** reduced PLC∂1PH-RFP levels at the membrane, indicative of PIP_2_ hydrolysis. **G)** Quantification of translocation in HEK293 cells. **P < 0.005 compared to pre-rapamycin condition, n = 20 cells per condition. Scale bar, 10 μm.

When cotransfected into human embryonic kidney 293 (HEK293) cells [28], FRB^PLF^-CFP localized to the plasma membrane (Figure 1A), Venus-FKBP12-Inp54p was localized to the cytoplasm (Figure 1B), and PLC∂1-PH-RFP was bound to the PIP_2_-rich plasma membrane (Figure 1C, quantified in Figure 1G), as expected. The apparent localization of the Venus-FKBP12-Inp54p construct to the plasma membrane at sites of cell-cell contact represents an artifact called “pseudolocalization” [29], and is not actual membrane localization. After treatment with 1 μM rapamycin, there was no change in membrane localization of the FRB^PLF^ domain (Figure 1D), but Venus-FKBP12-Inp54p translocated to the plasma membrane (Figure 1E) and hydrolyzed PIP_2_, as evidenced by displacement of PLC∂1-PH-RFP to the cytoplasm (Figure 1F). Furthermore, rapamycin reduced Gαq-coupled GPCR signaling (using 1 mM carbachol stimulation and calcium imaging as readout; data not shown). Lastly, rapamycin induced translocation of Venus-FKBP12-Inp54p to the plasma membrane in additional cell lines, including Rat1 fibroblasts, HeLa cells, and COS7 cells (data not shown).

### Targeting FRB^PLF^-CFP and Venus-FKBP12-Inp54p to peptidergic sensory neurons

**Figure 2:**
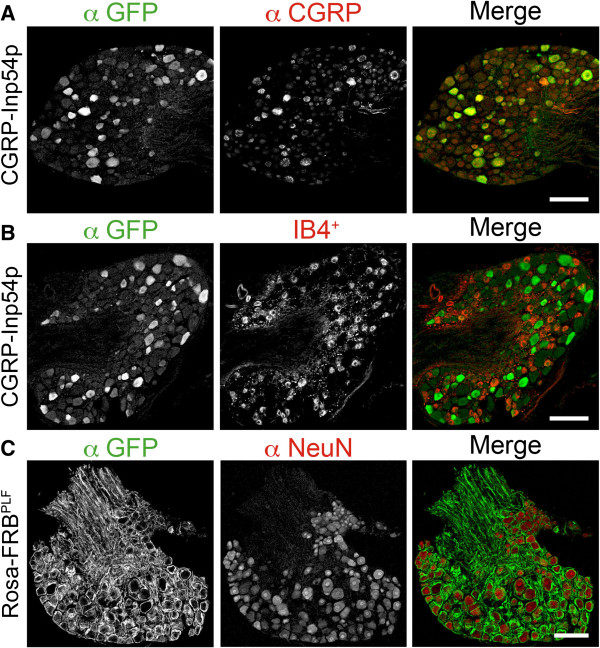
**CGRP-Inp54p and Rosa-FRB**^**PLF **^**knockin mice successfully target their respective knockin proteins in DRG neurons.** Sections from lumbar DRG from **A,B)** CGRP-Inp54p^+/−^ knockin mouse stained with antibodies to GFP (cross-reacts with Venus and CFP), CGRP and fluorescent IB4-binding. Venus-FKBP12-Inp54p was confined to cell bodies of CGRP^+^ neurons. **C)** FRB^PLF^-CFP mouse stained with antibodies to GFP and the pan-neuronal marker NeuN. FRB^PLF^-CFP was localized to the membrane and axons of all neurons. Representative of two animals per condition. Scale bar, 100 μm.

Small diameter sensory neurons in the DRG can be divided into peptidergic and nonpeptidergic subsets, with CGRP marking peptidergic neurons, and the plant lectin isolectin B4 (IB4) marking nonpeptidergic neurons [30]. The peptidergic subset responds to stimuli that evoke sensations of pain and itch, expresses the noxious heat receptor TRPV1, and can be genetically targeted by knocking genes into the CGRPα locus [23,24]. Thus, to examine the role of PIP_2_ in thermal nociception, we knocked Venus-FKBP12-Inp54p into the CGRPα locus. Heterozygous and homozygous CGRP-Inp54p mice were viable and fertile. Using immunohistochemistry, 87.9% of all CGRP-immunoreactive DRG neurons contained Venus-FKBP12-Inp54p protein (Figure 2A). In contrast, few (3.7%) nonpeptidergic IB4+ neurons contained Venus-FKBP12-Inp54p (Figure 2B). These findings were consistent with the limited overlap between CGRP-IR/CGRPα and IB4 [23,24,30,31], and indicate that Venus-FKBP12-Inp54p is primarily expressed in peptidergic sensory neurons.

The Rosa26 locus ubiquitously drives expression in all cell types, including DRG neurons [32,33]. Thus, we targeted FRB^PLF^-CFP to the plasma membrane, using a strong CMV early enhancer element and chicken beta-actin (CAG) promoter in the Rosa26 locus, to drive higher gene expression than the endogenous Rosa26 promoter alone [34]. Rosa-FRB^PLF^ heterozygous and homozygous mice were viable and fertile. Using immunohistochemistry, we found that FRB^PLF^-CFP was present on the plasma membrane of approximately 99% of all DRG neurons (Figure 2C). For all conditions, n > 500 neurons were counted from two animals.

### Rapamycin did not induce translocation of Venus-FKBP12-Inp54p from the cytoplasm to the plasma membrane in DRG neurons

**Figure 3:**
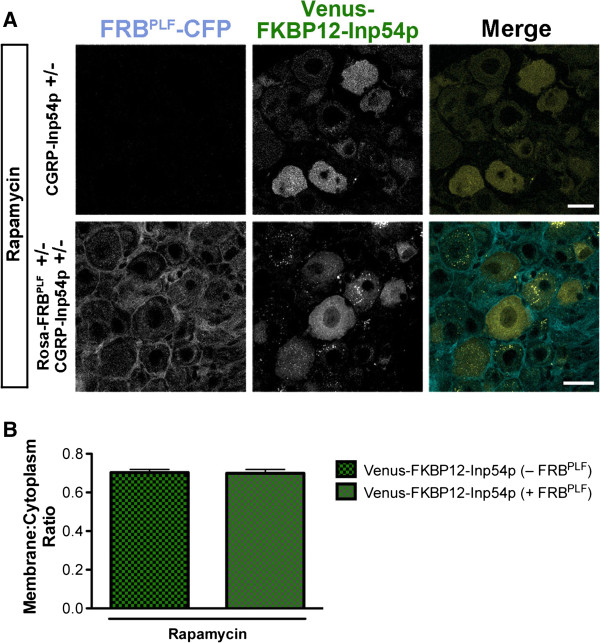
**Venus-FKBP12-Inp54p did not translocate to the membrane of DRG neurons after rapamycin treatment in knockin mice. A)** CGRP-Inp54p^+/−^ (top) and Rosa-FRB^PLF^/CGRP-Inp54p double heterozygous (bottom) mice were injected with rapamycin (i.t., three 1 nmol injections spaced evenly over 24 h, followed by paraformaldehyde perfusion shortly after the third injection). DRG were then dissected, sectioned, and mounted without immunostaining. **B)** Quantification of translocation. No significant difference in the membrane to cytoplasmic ratio of Venus-FKBP12-Inp54p in neurons containing FRB^PLF^ (n = 93 neurons) compared to neurons lacking FRB^PLF^ (n = 89 neurons). Scale bar, 20 μm.

We next crossed Rosa-FRB^PLF^ mice with CGRP-Inp54p mice to generate compound heterozygotes that expressed both components in peptidergic DRG neurons (Figure 3A). Intrathecal (i.t.) injection of rapamycin was previously used to dimerize mTOR-FKBP12 in DRG neurons *in vivo*[35,36]. In light of this information, we intrathecally injected CGRP-Inp54p^+/−^ (controls) and Rosa-FRB^PLF^/CGRP-Inp54p compound heterozygous mice with rapamycin (3 injections, spread over 24 h). Animals were perfused shortly after the final rapamycin injection to fix proteins that may have translocated. DRG were then dissected, cryo-sectioned, and mounted for imaging. Translocation of Venus-FKBP12-Inp54p was assessed by quantifying the location of endogenous Venus fluorescence without immunoenhancement, as we were unable to use antibodies to GFP/Venus because they cross-react to CFP, a highly related protein. Venus and CFP were present in our mice as single-copy knockins, making it difficult to see endogenous fluorescence without increasing gain, and explaining why signals were weak. Unfortunately, this rapamycin treatment did not induce translocation of Venus-FKBP12-Inp54p from the cytoplasm to the plasma membrane in controls or in double heterozygous mice (Figure 3A, quantified in Figure 3B), suggesting that this two-component system was non-functional in DRG neurons *in vivo*.

### Rapamycin treatment did not affect behavior in Rosa-FRB^PLF^/CGRP-Inp54p heterozygous mice *in vivo*

**Figure 4:**
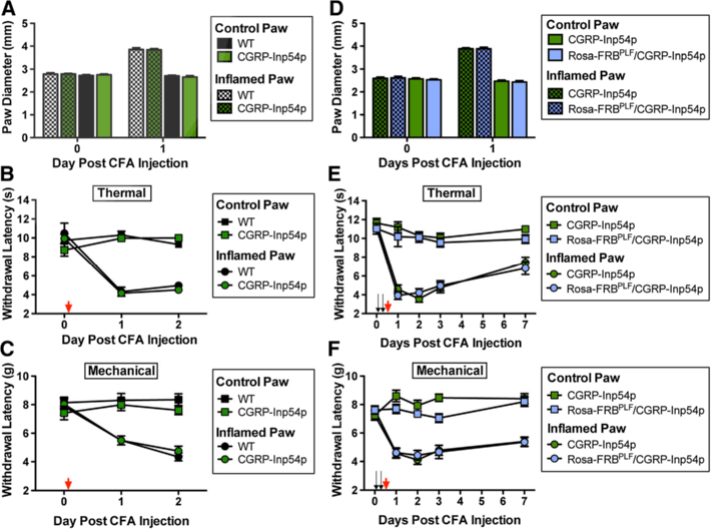
**Rapamycin treatment of Rosa-FRB**^**PLF**^**/CGRP-Inp54p mice did not affect hypersensitivity following inflammation. A-C)** Control experiments with CGRP-Inp54p^+/−^ mice. **A)** Edema, **B)** noxious thermal hyperalgesia and **C)** mechanical allodynia following intraplantar injection of CFA. No significant differences between genotypes before or after CFA injection. n = 12 male mice per genotype. **D-F)** Rosa-FRB^PLF^/CGRP-Inp54p double heterozygous mice. **D)** Edema, **E)** noxious thermal hyperalgesia and **F)** mechanical allodynia following intraplantar injection of CFA. Black arrows indicate i.t. rapamycin (1 nmol) injections, red arrows indicate intraplantar CFA injection. No significant differences between genotypes before or after CFA injection. n = 10 male mice per genotype.

We recently found that PIP_2_ levels in DRG at the time of inflammation enduringly affected thermal hypersensitivity, with preemptively reduced PIP_2_ levels correlating with an enduring reduction in thermal hypersensitivity [22]. Thus, we hypothesized that complete Freunds adjuvant (CFA)-induced thermal hypersensitivity would be blunted in Rosa-FRB^PLF^/CGRP-Inp54p mice if rapamycin caused a significant depletion of PIP_2_. Although we did not observe translocation in rapamycin-injected animals, we hypothesized that behavior might be a more sensitive readout of system performance than translocation. First, we performed control experiments with CGRP-Inp54^+/−^ mice to ensure that expression of this phosphatase alone had no effect on behavioral responses. We tested noxious thermal and mechanical sensitivity in wild-type (WT) and CGRP-Inp54p heterozygous littermates before and after inflaming one hindpaw with CFA, to model chronic inflammatory pain. We found that WT and CGRP-Inp54p^+/−^ mice showed no significant differences in paw diameter, thermal sensitivity or mechanical sensitivity before or after CFA injection (Figure 4A-C). Thus, expression of Venus-FKBP12-Inp54p in CGRPα-positive neurons did not alter nociceptive behavioral responses.

Next, we tested the effects of preemptive rapamycin i.t. injections on thermal and mechanical hypersensitivity in CGRP-Inp54p^+/−^ (control) mice and Rosa-FRB^PLF^/CGRP-Inp54p compound heterozygous mice. After baseline testing, we injected these mice twice with rapamycin (i.t.) at 6 hour intervals, then injected CFA into one hindpaw immediately after the second injection. CFA injections were successful, as evidenced by edema (Figure 4D), thermal hypersensitivity (Figure 4E) and mechanical allodynia (Figure 4F) in the inflamed paw, but not the control paws. However, there were no significant differences between control and experimental groups, further suggesting that this two-component system was non-functional in DRG neurons.

### Rapamycin treatment stabilized FRB^PLF^-CFP but did not induce translocation of Venus-FKBP12-Inp54p in cultured DRG neurons

**Figure 5:**
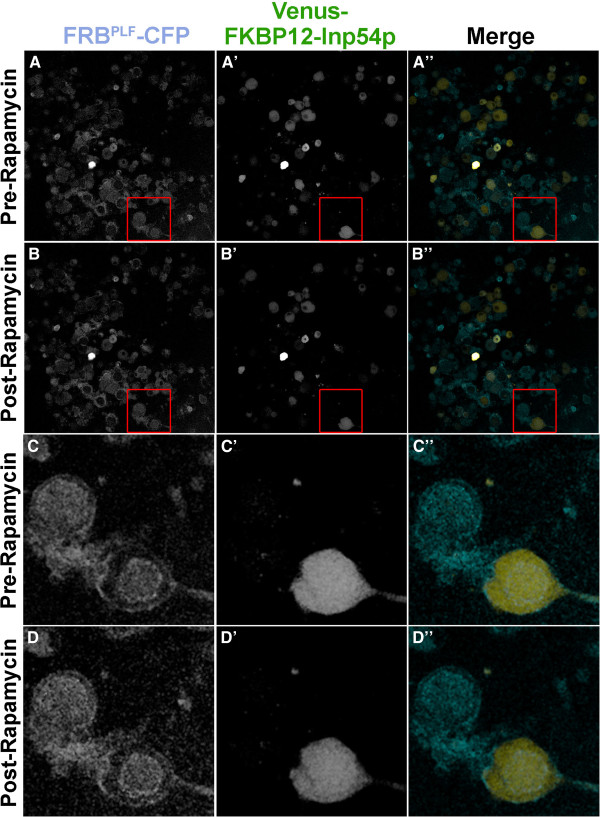
**Short-term rapamycin treatment does not induce translocation of Venus-FKBP12-Inp54p in cultured DRG neurons. A)** Cultured DRG neurons from male Rosa-FRB^PLF^/CGRP-Inp54p mice were plated for 24 hours. **B)** 1 μM rapamycin was applied for 10 minutes, and post-rapamycin images were taken. **C)** Close-up (5x zoom) of box region in **(A)** to show pre-rapamycin localization, and **D)** 5x zoom of box region in **(B)** to demonstrate the lack of Inp54p enrichment to FRB-tagged membrane after rapamycin treatment. **A-D** show FRB^PLF^-CFP localization, **A’-D’** demonstrate Venus-FKBP12-Inp54p localization, and the merge is shown in A”-D”.

**Figure 6:**
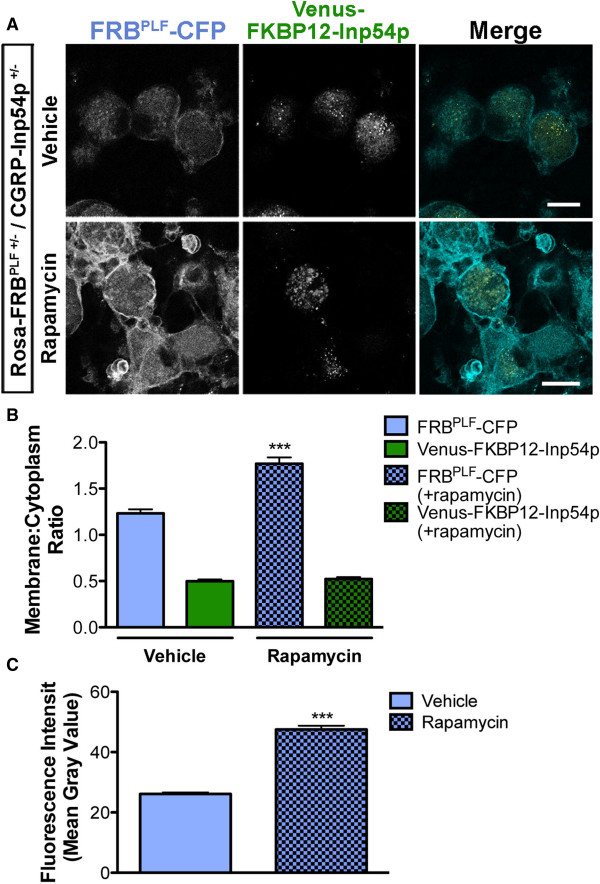
**Rapamycin stabilizes FRB**^**PLF**^**-CFP in cultured DRG neurons but does not induce translocation of Venus-FKBP12-Inp54p. A)** Confocal images of cultured DRG neurons from Rosa-FRB^PLF^/CGRP-Inp54p double heterozygous mice after culturing for 24 hours in presence of vehicle or 1 μM rapamycin. **B)** Quantification following vehicle/rapamycin treatment. While the ratio of FRB^PLF^-CFP increases after rapamycin treatment, this is due to **C)** an increase in FRB^PLF^-CFP fluorescence, rather than translocation. n = 86 neurons per condition ***P < 0.0001. Scale bar, 20 μm.

We next evaluated whether rapamycin could induce translocation of Venus-FKBP12-Inp54p to the plasma membrane in cultured DRG neurons. To test this, we cultured DRG neurons from Rosa-FRB^PLF^/CGRP-Inp54p double heterozygous mice, treated cells for 10 min with vehicle or 1 μM rapamycin, then imaged both components (CFP and Venus fluorescence) using confocal microscopy. As with our *in vivo* studies above, rapamycin treatment did not induce translocation of Venus-FKBP12-Inp54p to the plasma membrane (Figure 5). We then treated cultured DRG neurons from Rosa-FRB^PLF^/CGRP-Inp54p double heterozygous for longer periods of time. Unfortunately, we still were unable to detect translocation even after 24 hours (Figure 6A-B) or 48 hours (data not shown). Notably however, prolonged treatment with rapamycin stabilized FRB^PLF^-CFP, as evidenced by increased fluorescence signal after 24 hours (Figure 6A-B, quantified in Figure 6C; all gain settings the same). The FRB^PLF^ domain can be stabilized within hours after dimerizing with endogenous FKBP12 [25,37].

**Figure 7:**
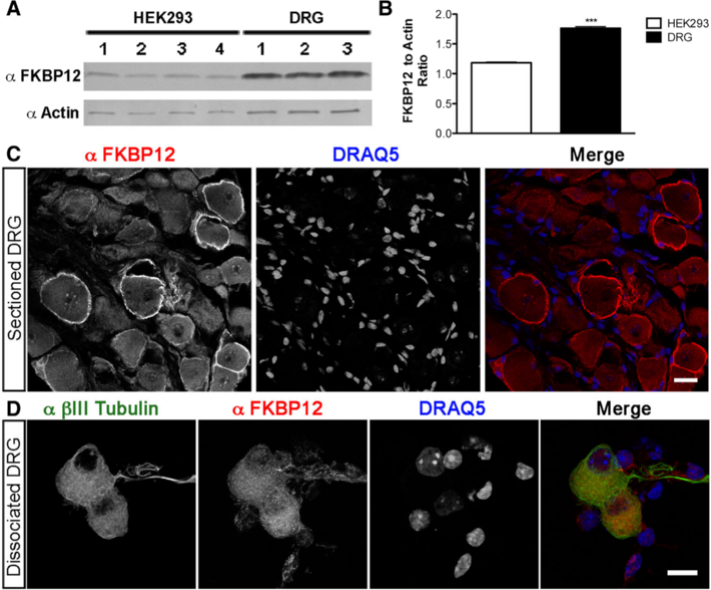
**Endogenous FKBP12 protein levels are significantly higher in DRG neurons when compared to HEK293 cells. A)** Western blot of HEK293 cell lysates (from 4 separate cultures) and DRG lysates (dissected from three 8-week old WT mice) probed with antibodies to FKBP12 and beta-actin. **B)** Quantification of the FKBP12 protein levels, normalized to actin loading control. ***p < 0.0001. **C)** Section from WT DRG immunostained with antibodies to FKBP12 and counterstained with DRAQ5 to mark nuclei. Scale bar, 20 μm. **D)** Dissociated neurons stained with anti-βIII Tubulin (a neuron-specific marker), anti-FKBP12, and DRAQ5. Endogenous FKBP12 is present at much higher levels in neurons compared to non-neuronal cells (tubulin^-^, DRAQ5^+^ cells; p < 0.0001). Scale bar, 10 μm.

Our data suggested that DRG neurons might contain high levels of endogenous FKBP12 that compete with Venus-FKBP12-Inp54p for binding to FRB^PLF^-CFP. Moreover, we hypothesized that HEK293 cells might express lower levels of endogenous FKBP12 than DRG neurons, given that Venus-FKBP12-Inp54p did translocate to the membrane in HEK293 cells expressing FRB^PLF^-CFP (Figure 1). Indeed, we found that endogenous FKBP12 levels were significantly higher in DRG when compared to HEK293 cells (Figure 7A-B). Although the level of FKBP12 is only 1.5 higher in total DRG lysate (Figure 7B), this is likely an underestimation of FKBP12 in DRG neurons due to dilution by non-neuronal DRG cells, as FKBP12 is expressed more highly in neurons than non-neuronal surrounding cells of the DRG (Figure 7C-D). COS7 cells also contained low levels of FKBP12 (data not shown), possibly explaining why Venus-FKBP12-Inp54p translocated to the plasma membrane in this cell line as well (see above).

To delineate the localization of FKBP12, we immunostained DRG sections from WT animals with antibodies to FKBP12. FKBP12 was found throughout the cytoplasm in all neurons, and was often concentrated at the membrane in large diameter DRG neurons (Figure 7C). Notably, the satellite cells that surround DRG neurons (marked by DRAQ5-positive nuclei) contained lower levels of FKBP12 (Figure 7C). Likewise, in cultures of dissociated DRG, high levels of FKBP12 were detected in βIII Tubulin^+^ neurons (a neuronal-specific marker), while βIII Tubulin^-^, DRAQ5^+^ cells had lower levels of FKBP12 (Figure 7D; quantified by image intensity analysis; p < 0.0001, data not shown). Thus, FKBP12 was present at high levels in DRG neurons, and at low levels in non-neuronal cells in the DRG.

## Discussion

**Figure 8:**
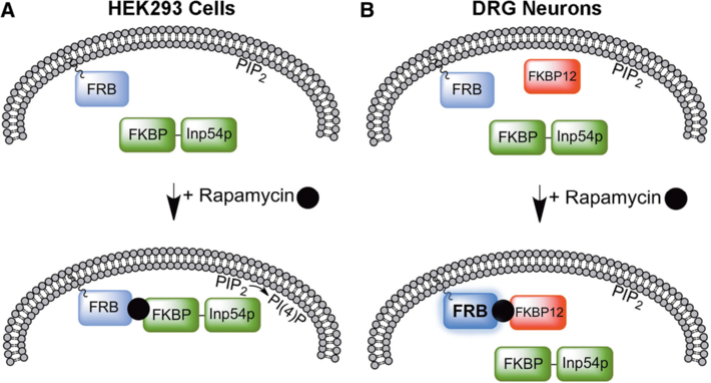
**Model: endogenous FKBP12 limits use of rapamycin-induced translocation in DRG neurons but not HEK293 cells. A)** In HEK293 cells, rapamycin promotes dimerization of membrane-tethered FRB^PLF^-CFP with Venus-FKBP12-Inp54p. Once at the membrane, Venus-FKBP12-Inp54p hydrolyzes PIP_2_ to PI(4)P. **B)** DRG neurons contain high levels of endogenous FKBP12 in the cytoplasm and, in some neurons, on the membrane. Rapamycin stabilizes FRB^PLF^-CFP (bold), but does not promote translocation of Venus-FKBP12-Inp54p to the membrane. These data suggest that rapamycin preferentially facilitates dimerization of FRB^PLF^-CFP with endogenous FKBP12 and occludes dimerization with Venus-FKBP12-Inp54p. Venus-FKBP12-Inp54p remains in the cytoplasm, unable to access PIP_2_ in the membrane.

We successfully generated two knockin mice that each expressed components of the rapamycin-inducible PIP_2_ depletion system. FRB^PLF^-CFP and Venus-FKBP12-Inp54p were expressed in the appropriate cell types and each of these proteins was targeted to the correct subcellular location (membrane and cytoplasm, respectively). While Venus-FKBP12-Inp54p translocated to the membrane in cell lines expressing FRB^PLF^-CFP, we were unable to detect rapamycin-induced translocation of these components in DRG neurons *in vitro* or *in vivo*. Furthermore, rapamycin treatment of double heterozygous mice did not alter thermal sensitivity as we would have expected if the system had worked *in vivo*. While this chemically-induced translocation tool is widely used for manipulation in cell lines, our data collectively suggest that high levels of endogenous FKBP12 limit its functionality in DRG neurons (Figure 8).

While rapamycin did not induce translocation in DRG neurons, it did enhance CFP-FRB^PLF^ protein fluorescence intensity, suggesting that rapamycin interacted with FRB^PLF^ and promoted dimerization to endogenous FKBP12. The FRB domain mutation used in our Rosa-FRB mouse consists of three point mutations: K2095P, T2098L, and W2101F [26]. These mutations allow for the use of rapamycin analogs that do not cross-react with the wildtype, endogenous FRB domain of mTOR. One of these mutations (T2098L) is responsible for protein destabilization, and this destabilized FRB mutant has been previously used for successful dimerization *in vivo*[25,26,37]. Interestingly, this destabilization is extended to proteins fused to the FRB^PLF^ mutant, such as fluorescent tags, and is reversed upon FKBP12-rapamycin-FRB^PLF^ complex formation, with a half-maximal result approximately 8 hours after rapamycin treatment [25,37]. The fact that FRB^PLF^-CFP protein levels were increased demonstrates that rapamycin was in fact reaching its intended target in DRG neurons. However, Venus-FKBP12-Inp54p failed to translocate, suggesting that endogenous FKBP12 could be responsible for this stabilization. Indeed, DRG neurons contain higher levels of endogenous FKBP12 than HEK293 cells (Figure 7). Endogenous FKBP12 in DRG neurons could potentially out-compete Venus-FKBP12-Inp54p for binding to FRB^PLF^, and thus prevent Venus-FKBP12-Inp54p from translocating to the membrane. Alternatively, the levels of mTOR, the protein that contains the endogenous FRB domain, could have a similar effect on sequestering the transgenic Venus-FKBP12-Inp54p protein away from the membrane-tagged FRB^PLF^ domain. The rapamycin analog C20-Marap binds to FRB^PLF^ without interacting with endogenous mTOR [26]; therefore, this compound could potentially be used to rule out the role of endogenous mTOR as a source of translocation inhibition. However, it should be emphasized that use of rapalogs would not overcome the problem we identified, namely interaction of FRB with endogenous FKBP12.

Overexpression of Inp54p in cell lines can lead to loss of cell adhesion, induction of membrane blebbing, and ultimately cell death [38,39]. Expression after transfection in cultured cells tends to be on the timescale of a few days, whereas these mice express Venus-FKBP12-Inp54p throughout the life of the animal. It is possible that some compensation occurs when Inp54p is expressed over longer time scales.

In addition to elevated levels of endogenous FKBP12, other factors might limit rapamycin-induced translocation *in vivo*. Based on our experiments with FRB^PLF^, FKBP12-Inp54p, and PH constructs in cell lines, we noticed that the ratio between the three proteins varied highly between individual cells. Indeed, others similarly noted that the ratio of each component was critical for experimental success [29,40]. Therefore, the ratio of FRB^PLF^-CFP to Venus-FKBP12-Inp54p in our mice might be suboptimal for translocation.

Given the widespread use of rapamycin-induced dimerization to study biological processes in cell lines, it is perhaps remarkable to note that there is only one publication describing the use of rapamycin-induced heterodimerization *in vivo*[25]. Notably, in this study Stankunas et al. use rapamycin analogs to dimerize and stabilize a cytoplasmically localized FRB^PLF^ fusion protein with endogenous FKBP12 (also cytoplasmic) in cultured mouse embryonic fibroblasts (MEFs) and embryonic forelimb tissue. They did not attempt to dimerize FRB^PLF^ with an engineered FKBP-domain to translocate protein, nor did their study utilize this system in neurons. Karpova and colleagues used a FKBP homodimerization system, consisting of two mutated FKBP domains (F36V) to regulate neurotransmission in transgenic mice [41]. Thus, it appears that no lab has successfully found a way to inducibly heterodimerize engineered FRB with engineered FKBP *in vivo*.

## Conclusions

While rapamycin-induced translocation is highly effective for studying signaling events in a temporally-controlled manner in cell lines, our results—taken together with the lack of published reports of rapamycin-induced translocation *in vivo*—suggest that there are limitations that prevent the adaptation of this system for use in neurons *in vitro* and *in vivo*. Supporting this hypothesis, we found that brain lysates and DRG lysates had equally high levels of FKBP12, and it has previously been noted that high levels of FKBP12 mRNA are found throughout nervous tissue, including cerebral cortex and hippocampus, compared to non-neuronal tissue [42]. Therefore, elevated levels of endogenous FKBP12 could restrict the utilization of rapamycin-induced translocation in neuronal cells in general. To our knowledge, this issue has not been previously identified or raised. Our study could thus spur the development of new reagents, like novel rapalogs that interact with engineered versions of FKBP12 but not endogenous FKBP12. Such reagents, when combined with FRB mutants that do not interact with endogenous mTOR, could allow greater adoption of this dimerization system *in vitro* and *in vivo*.

## Methods

All procedures and behavioral experiments involving vertebrate animals were approved by the Institutional Animal Care and Use Committee at the University of North Carolina at Chapel Hill.

### DNA plasmid constructs

Constructs for rapamycin-induced PIP_2_ depletion in HEK293 cells were obtained from Ken Mackie (University of Indiana), Tamas Balla (NICHD, Bethesda, MD USA) and Tobias Meyer (Stanford, Stanford, CA USA). The RFP-tagged PH domain of rat PLC∂1 was a kind gift from Ken Mackie. The CFP-tagged FRB domain was tethered to the plasma membrane using the first 20 amino acids of the human GAP43, as described in Várnai et al. (2006), was obtained from Tamas Balla, and cloned into pcDNA3.1(+). The FKBP-Inp54p yeast 5-phosphatase construct was a gift from Tobias Meyer, cloned into pcDNA3.1(+), and modified with a Venus fluorescent protein tag.

### Cell culture and live-imaging

HEK293 cells were grown on glass bottom cell culture dishes (MatTek; P35G-0-10-C) in Dulbecco’s Modified Eagle Medium (DMEM, Sigma) supplemented with 10% fetal bovine serum, 100 U/ml penicillin, and 100 μg/ml streptomycin. Cells were transfected with 4 μl Lipofectamine 2000 (Invitrogen) and 1 μg total DNA per culture dish in Opti-MEM (Gibco) for 2 hours, at which point media was replaced with the supplemented DMEM. After 16–24 hours, supplemented DMEM was replaced with Hank’s Balanced Salt Solution (HBSS Gibco 14025, supplemented with 9 mM HEPES, 11 mM D-glucose, 0.1% fatty-acid free BSA, pH 7.3) warmed to 37°C. After baseline imaging, HBSS was replaced with HBSS containing 1 μM rapamycin (Calbiochem). Each plate was imaged on a Leica TCS confocal microscope using a 40x objective, and maintained at 37°C throughout the imaging session using a heated stage attachment. Cells were treated for 10 minutes, at which point a final post-rapamycin image was taken. Membrane to cytoplasm ratio in pre- and post-rapamycin-treated cells was measured in cells expressing all three constructs, using NIH ImageJ software.

### Generation of FRB^PLF^-CFP and Venus-FKBP12-Inp54p knockin mice

The GAP43-FRB^PLF^-CFP construct containing three point mutations of the FRB domain (K2095P, T2098L, and W2101F) was cloned into the Rosa26 targeting construct. This insert was placed under the control of the CAG promoter, and the entire CAG-GAP43-FRB^PLF^-CFP insert was followed by a self-excising neomycin resistance cassette (ACN) [43]. CGRP targeting was accomplished by recombineering of *Calca* targeting arms from a C57BL/6-derived bacterial artificial chromosome (BAC; RP24-136021). The start codon, located in exon 2, is common to CGRPα and calcitonin and was replaced with an AscI site to facilitate cloning. The Venus-FKBP12-Inp54p construct described above was cloned into this CGRP targeting construct, without an external promoter, but with the ACN cassette. Successful targeting of embryonic stem cells by homologous recombination was identified with Southern blot hybridization, using probes that flanked the 5’ and 3’ arms of the targeting constructs, as well as an internal neomycin probe. Chimeric mice were produced by blastocyst injection, and mated to C57BL/6 mice to establish the line.

Transgenic mice were identified by PCR amplification of genomic DNA with specific primers. CGRP2 (5’ CAGCTCCCTGGCTTTCATCTGC), CGRP (5’ AAATGTCGGGGAGTCACAGGC), and EGFP2 (5’ CCGTAGGTCAGGGTGGTCACGAGG) were used to evaluate wildtype and/or knockin bands for CGRP knockin mice. Internal CFP primers (5’ CGATGAGATGTGGCATGAAGG and 5’ CCGTCGTCCTTGAAGAAGATGG) were used to detect the presence of the Rosa-FRB^PLF^-CFP knockin allele.

### Neuronal dissociation and imaging

Male WT and Rosa-FRB^PLF^/CGRP-Inp54p mice (3–4 weeks old) were decapitated without anesthesia, and the DRG were dissected into ice-cold Hank’s Balanced Salt Solution (HBSS; Gibco,14175-095), and dissociated using collagenase (1 mg/mL; Worthington, CLS1) and dispase (5 mg/mL; Gibco, 17105–041) dissolved in HBSS. Neurons were plated onto coverslips coated with 0.1 mg/mL poly-D-lysine (Sigma P0899) and 5 μg/mL laminin (Sigma, L2020), and cultured in Neurobasal-A medium (Invitrogen, 10888022), supplemented with B-27 Supplement (Gibco, 17504–044), L-glutamine (Gibco, 25030–081), and penicillin-streptomycin (Gibco, 15140–122), and 5% fetal bovine serum. WT neurons were grown with no fetal bovine serum, but with the addition of 0.25 ng/mL nerve growth factor, and 0.5 ng/mL glial derived neurotrophic factor. WT neurons were fixed with 4% paraformaldeyde (warmed to 37°C) at 24 hours *in vitro* and then immunostained. For Rosa-FRB^PLF +/−^/CGRP-Inp54p^+/−^ neurons, rapamycin was added to neuronal culture medium at a final concentration of 1 μM in half of the wells, and cultured for 24 and 48 hours. This allowed for comparison of treated neurons to vehicle controls. Dissociated neuronal cultures were prepared as described above, and fixed at 24 hours *in vitro* with 4% PFA warmed to 37°C for 30 min. Neurons were washed with PBS to remove fixative, mounted on slides, and imaged on a Leica TCS confocal microscope. CFP was excited with a 458 nm laser and detected with emission settings of 465–505 nm, and Venus was excited using a 514 nm laser and detected with emission settings of 528–587 nm.

### Immunohistochemistry

For DRG tissue sections, male mice 4–6 weeks were injected intraperitoneally with pentobarbital, and perfused with 4% PFA in 0.1 M phosphate buffer, pH 7.4. Lumbar DRG (L2-L6) were dissected and post-fixed for 2 hours in 4% PFA. The DRG were subsequently cryoprotected in 30% sucrose, 0.1 M phosphate buffer, pH 7.3 at 4°C for 24 h, frozen in OCT TissueTek, cryosectioned at 20 μm, mounted on Superfrost Plus slides, and stored at −20°C until use. Tissue was rehydrated and washed in PBS to remove OCT embedding compound, and either coverslipped immediately, or prepared for immunohistochemistry (IHC). DRG sections and dissociated neurons were permeablized and blocked in TBS-Tx (0.05 M Tris, 2.7% NaCl, 0.3% Triton-X 100, pH 7.6) containing 10% normal goat serum (NGS) for 1 hr at room temperature. Sections were incubated overnight at 4°C with primary antibodies in TBS-Tx/10%NGS, washed, incubated at room temperature for 2 hours with secondary antibodies in TBS-Tx/10%NGS, washed, and mounted with Fluorogel (Biomeda). Primary antibodies used were chicken anti-GFP (1:500; Aves Labs, GFP-1020), rabbit anti-CGRP (1:750; Peninsula, T-4032), mouse anti-NeuN (1:200, Millipore), and rabbit anti-FKBP12 (1:125, Abcam) in TBS-Tx/10% NGS. Secondary antibodies include goat anti-chicken Alexa fluor 488 (1:2000, Invitrogen), goat anti-Rabbit Alexa fluor 633 (1:2000, Invitrogen), goat anti-mouse Alexa fluor 633 (1:2000, Invitrogen), Alexa fluor 633 conjugated to IB4 (1:1000, Invitrogen), and DRAQ5 (1:10000, Axxora).

### Biochemistry

Cells were lysed in ice-cold RIPA buffer: Tris, pH 7.4 (50 mM), Triton-X (1%), Sodium Dexocholate (0.25%), SDS (0.1%), EDTA (1 mM), NaCl (150 mM), Complete Protease Inhibitory Cocktail (1x, Roche), PMSF (1 mM). HEK293 cells were scraped from a 10 cm dish in RIPA buffer, and DRG were homogenized in RIPA buffer prior to spindown. Lysates were placed on ice for 20 minutes, then spun at 13200 rpm for 10 minutes. Supernatent was removed and used for Western blot analysis. Twenty micrograms of protein were loaded per lane in a 4-15% Tris gel, transferred onto PVDF membrane, blocked with 5% milk in TBS-T, incubated with primary antibodies (rabbit anti-FKBP12, Abcam, 1:1000; mouse anti-actin 1:3000) in 3% milk in TBS-T at 4°C overnight, washed in TBS-T, incubated with secondary antibodies (donkey anti-rabbit 800, Odyssey, 1:20000; donkey anti-mouse 680, Odyssey, 1:20000) for 1 hour at room temperature, washed, and developed.

### Drug administration for tissue extraction

Mice that were heterozygous for CGRP-Inp54p, or heterozygous for both Rosa-FRB^PLF^ and CGRP-Inp54p, received three intrathecal injections (each containing 1 nmol rapamycin, dissolved in saline to a total volume of 5 uL) over the course of 24 hours. Mice were then perfused and dissected, as described above, two hours after the second round of injections.

### Behavior

Male 3- to 4-month-old CGRP-Inp54p^+/−^ and WT littermates (n = 12 per genotype), or Rosa-FRB^PLF^/CGRP-Inp54p and CGRP-Inp54p heterozygous littermates (n = 10 per genotype), were acclimated to the testing apparatuses and experimenter for 2 days prior to behavioral testing. The experimenter was blind to genotype throughout the experiment. For CGRP-Inp54p^+/−^ and WT controls, baseline thermal and mechanical responses were monitored using Hargreaves and Von Frey apparatuses (as previously described in [22]) prior to intraplantar injection with complete Freund’s adjuvant (CFA) into the left hindpaw. Behavior testing for both thermal and mechanical sensitization was carried out on subsequent days, as described previously [22]. Rosa-FRB^PLF^/CGRP-Inp54p double heterozygous and CGRP-Inp54p^+/−^ littermate controls were used to determine the extent of rapamycin-induced depletion of PIP_2_ in the reduction of pain sensitivity *in vivo*. For this, each mouse received two intrathecal injections of rapamycin (1 nmol in 5 μl), one just after baseline measurement, and one just prior to CFA injection into the hindpaw 6 hours later, and behavioral testing was carried out on subsequent days.

## Competing interests

The authors declare that they have no competing interests in this study.

## Authors’ contributions

JCB, SBS, and MJZ conceived of and designed the experiments used in this study. JCB and BJF carried out the experiments presented in this manuscript, while SBS and JER performed experiments leading to the findings described in this manuscript. JCB and MJZ wrote the manuscript, and all authors approved the submission of this work.

## References

[B1] Crabtree GR Schreiber SL Three-part inventions: intracellular signaling and induced proximity Trends Biochem Sci 1996 21 418 422 10.1016/S0968-0004(96)20027-1 8987395

[B2] Jin L Zeng H Chien S Otto K Richard RE Emery DW Blau A In vivo selection using a cell-growth switch Nature Gen 2000 26 64 66 10.1038/79194 10973250

[B3] Suh B-C Inoue T Meyer T Hille B Rapid chemically induced changes of PtdIns(4,5)P2 gate KCNQ ion channels Science 2006 314 1454 1457 10.1126/science.1131163 16990515 PMC3579521

[B4] Varnai P Thyagarajan B Rohacs T Balla T Rapidly inducible changes in phosphatidylinositol 4,5-bisphosphate levels influence multiple regulatory functions of the lipid in intact living cells J Cell Biol 2006 175 377 382 10.1083/jcb.200607116 17088424 PMC2064515

[B5] Xu T Johnson CA Gestwicki JE Kumar A Conditionally controlling nuclear trafficking in yeast by chemical-induced protein dimerization Nat Protocol 2010 5 1831 1843 10.1038/nprot.2010.141 PMC4976631 21030958

[B6] Putyrski M Schultz C Switching Heterotrimeric G Protein Subunits with a Chemical Dimerizer Chem Biol 2011 18 1126 1133 10.1016/j.chembiol.2011.07.013 21944751

[B7] Majerus PW Ross TS Cunningham TW Caldwell KK Jefferson AB Bansai VS Recent insights in phosphatidylinositol signaling Cell 1990 63 459 465 10.1016/0092-8674(90)90442-H 2225061

[B8] Suh B-C Hille B PIP2 is a necessary cofactor for ion channel function: how and why? Annu Rev Biophys 2008 37 175 195 10.1146/annurev.biophys.37.032807.125859 18573078 PMC2692585

[B9] McLaughlin S Wang J Gambhir A Murray D PIP(2) and proteins; interactions, organization, and information flow Annu Rev Biophys Biomol Struct 2002 31 151 175 10.1146/annurev.biophys.31.082901.134259 11988466

[B10] Szentpetery Z Balla A Kim Y Lemmon M Balla T Live cell imaging with protein domains capable of recognizing phosphatidylinositol 4,5-bisphosphate; a comparative study BMC Cell Biol 2009 10 67 10.1186/1471-2121-10-67 19769794 PMC2755470

[B11] Várnai P Balla T Visualization of Phosphoinositides That Bind Pleckstrin Homology Domains: Calcium- and Agonist-induced Dynamic Changes and Relationship to Myo-[3H]inositol-labeled Phosphoinositide Pools J Cell Biol 1998 143 501 510 10.1083/jcb.143.2.501 9786958 PMC2132833

[B12] Stauffer T Ahn S Meyer T Receptor-induced transient reduction in plasma membrane PtdIns(4,5)P2 concentration monitored in living cells Curr Biol 1998 8 343 346 10.1016/S0960-9822(98)70135-6 9512420

[B13] Gamper N Shapiro M Regulation of ion transport proteins by membrane phosphoinositides Nat Rev Neurosci 2007 8 921 934 17971783 10.1038/nrn2257

[B14] Zaika O Zhang J Shapiro M Combined phosphoinositide and Ca2+ signals mediating receptor specificity toward neuronal Ca2+ channels J Biol Chem 2011 286 830 841 10.1074/jbc.M110.166033 21051544 PMC3013042

[B15] Chuang HH Prescott ED Kong H Shields S Jordt SE Basbaum AI Chao MV Julius D Bradykinin and nerve growth factor release the capsaicin receptor from PtdIns(4,5)P2-mediated inhibition Nature 2001 411 957 962 10.1038/35082088 11418861

[B16] Prescott E Julius D A modular PIP2 binding site as a determinant of capsaicin receptor sensitivity Science 2003 300 1284 1288 10.1126/science.1083646 12764195

[B17] Lukacs V Thyagarajan B Varnai P Balla A Balla T Rohacs T Dual regulation of TRPV1 by phosphoinositides J Neurosci 2007 27 7070 7080 10.1523/JNEUROSCI.1866-07.2007 17596456 PMC6672228

[B18] Rohacs T Thyagarajan B Lukacs V Phospholipase C mediated modulation of TRPV1 channels Mol Neurobiol 2008 37 153 163 10.1007/s12035-008-8027-y 18528787 PMC2568872

[B19] Kim A Tang Z Liu Q Patel K Maag D Geng Y Dong X Pirt, a phosphoinositide-binding protein, functions as a regulatory subunit of TRPV1 Cell 2008 133 475 485 10.1016/j.cell.2008.02.053 18455988 PMC2605970

[B20] Klein RM Ufret-Vincenty CA Hua L Gordon SE Determinants of molecular specificity in phosphoinositide regulation. Phosphatidylinositol (4,5)-bisphosphate (PI(4,5)P2) is the endogenous lipid regulating TRPV1 J Biol Chem 2008 283 26208 26216 10.1074/jbc.M801912200 18574245 PMC2533779

[B21] Daniels R Takashima Y McKemy D Activity of the neuronal cold sensor TRPM8 is regulated by phospholipase C via the phospholipid phosphoinositol 4,5-bisphosphate J Biol Chem 2009 284 1570 1582 19019830 10.1074/jbc.M807270200 PMC2615505

[B22] Sowa N Street S Vihko P Zylka M Prostatic acid phosphatase reduces thermal sensitivity and chronic pain sensitization by depleting phosphatidylinositol 4,5-bisphosphate J Neurosci 2010 30 10282 10293 10.1523/JNEUROSCI.2162-10.2010 20685973 PMC2920622

[B23] McCoy E Taylor-Blake B Zylka M CGRPα-expressing sensory neurons respond to stimuli that evoke sensations of pain and itch PLoS One 2012 7 e36355 10.1371/journal.pone.0036355 22563493 PMC3341357

[B24] McCoy ES Taylor-Blake B Street SE Pribisko AL Zheng J Zylka MJ Peptidergic CGRPalpha Primary Sensory Neurons Encode Heat and Itch and Tonically Suppress Sensitivity to Cold Neuron 2013 78 138 151 10.1016/j.neuron.2013.01.030 23523592 PMC3628403

[B25] Stankunas K Bayle JH Gestwicki JE Lin YM Wandless TJ Crabtree GR Conditional protein alleles using knockin mice and a chemical inducer of dimerization Mol Cell 2003 12 1615 1624 10.1016/S1097-2765(03)00491-X 14690613

[B26] Bayle J Grimley J Stankunas K Gestwicki J Wandless T Crabtree G Rapamycin analogs with differential binding specificity permit orthogonal control of protein activity Chem Biol 2006 13 99 107 10.1016/j.chembiol.2005.10.017 16426976

[B27] Schmidt-Michels M Edwards P Oestricher A Gispen W Colchicine effect on B-50/GAP43 phosphoprotein localization in rat dorsal root ganglion explants Neurosci Lett 1989 97 285 290 10.1016/0304-3940(89)90612-5 2717063

[B28] Graham F Smiley J Russell W Nairn R Characteristics of a human cell line transformed by DNA from human adenovirus type 5 J Gen Virol 1977 36 59 74 10.1099/0022-1317-36-1-59 886304

[B29] Balla T Várnai P Visualizing cellular phosphoinositide pools with GFP-fused protein modules Sci STKE 2002 125 pl3 11917154 10.1126/stke.2002.125.pl3

[B30] Zylka M Sowa N Taylor-Blake B Twomey M Herrala A Voikar V Vihko P Prostatic acid phosphatase is an ectonucleotidase and suppresses pain by generating adenosine Neuron 2008 60 111 122 10.1016/j.neuron.2008.08.024 18940592 PMC2629077

[B31] Cavanaugh DJ Lee H Lo L Shields SD Zylka MJ Basbaum AI Anderson DJ Distinct subsets of unmyelinated primary sensory fibers mediate behavioral responses to noxious thermal and mechanical stimuli Proc Natl Acad Sci 2009 106 9075 9080 10.1073/pnas.0901507106 19451647 PMC2683885

[B32] Soriano P Generalized lacZ expression with the ROSA26 Cre reporter strain Nat Genet 1999 21 70 71 10.1038/5007 9916792

[B33] Stirling LC Forlani G Baker MD Wood JN Matthews EA Dickenson AH Nassar MA Nociceptor-specific gene deletion using heterozygous NaV1.8-Cre recombinase mice Pain 2005 113 27 36 10.1016/j.pain.2004.08.015 15621361

[B34] Madisen L Zwingman T Sunkin S Oh S Zariwala H Gu H Ng L Palmiter R Hawrylysz M Jones A A robust and high-throughput Cre reporting and characterization system for the whole mouse brain Nat Neurosci 2009 13 151 175 10.1038/nn.2467 PMC2840225 20023653

[B35] Price T Rashid M Millecamps M Sanoja R Entrena J Cervero R Decreased Nociceptive Sensitization in Mice Lacking the Fragile X Mental Retardation Protein: Role of mGluR1/5 and mTOR J Neurosci 2007 27 13959 13967 10.1523/JNEUROSCI.4383-07.2007 PMC2206543 18094233

[B36] Geranton SM Jimenez-Diaz L Torsney C Tochiki KK Stuart SA Leith JL Lumb BM Hunt SP A rapamycin-sensitive signaling pathway is essential for the full expression of persistent pain states J Neurosci 2009 29 15017 15027 10.1523/JNEUROSCI.3451-09.2009 19940197 PMC2830115

[B37] Stankunas K Bayle JH Havranek JJ Wandless TJ Baker D Crabtree GR Gestwicki JE Rescue of degradation-prone mutants of the FK506-rapamycin binding (FRB) protein with chemical ligands Chembiochem 2007 8 1162 1169 10.1002/cbic.200700087 17525916

[B38] Azuma T Koths K Flanagan L Kwiatkowski D Gelsolin in Complex with Phosphatidylinositol 4,5-Bisphosphate Inhibits Caspase-3 and −9 to Retard Apoptotic Progression J Biol Chem 2000 275 3761 3766 10.1074/jbc.275.6.3761 10660524

[B39] Raucher D Stauffer T Chen W Shen K Guo S Phosphatidylinositol 4,5-bisphosphate functions as a second messenger that regulates cytoskeleton-plasma membrane adhesion Cell 2000 100 221 228 10.1016/S0092-8674(00)81560-3 10660045

[B40] Komatsu T Kukelyansky I McCaffery JM Ueno T Varela LC Inoue T Organelle-specific, rapid induction of molecular activities and membrane tethering Nat Methods 2010 7 206 208 10.1038/nmeth.1428 20154678 PMC2860863

[B41] Karpova AY Tervo DG Gray NW Svoboda K Rapid and reversible chemical inactivation of synaptic transmission in genetically targeted neurons Neuron 2005 48 727 735 10.1016/j.neuron.2005.11.015 16337911

[B42] Su A Wiltshire T Batalov S Lapp H Ching K Block D Zhang J Soden R Hayakawa M Kreiman G A gene atlas of the mouse and human protein-encoding transcriptomes Proc Natl Acad Sci USA 2004 101 6062 6067 10.1073/pnas.0400782101 15075390 PMC395923

[B43] Bunting M Bernstein K Greer J Capecchi M Thomas K Targeting genes for self-excision in the germ line Genes & Dev 1999 13 1524 1528 10.1101/gad.13.12.1524 10385621 PMC316811

